# Monitoring the detrimental impact of congested training periods on the strength levels and landing forces of young female aerobic gymnastics

**DOI:** 10.1016/j.heliyon.2024.e34609

**Published:** 2024-07-18

**Authors:** Dong Ma, Kezhan Zhao, Rui Miguel Silva, Ke Wang, Qi Xu, Zijian Zhao

**Affiliations:** a Gdansk University of Physical Education and Sport, 80-336, Gdańsk, Gdańsk, Poland; b Xi’an Yixin Middle School, 710082, Xi'an, China; c Escola Superior Desporto e Lazer, Instituto Politécnico de Viana do Castelo, Rua Escola Industrial e Comercial de Nun’Álvares, 4900-347, Viana do Castelo, Viana do Castelo, Portugal; d Sport Physical Activity and Health Research & Innovation Center, 4900-347, Viana do Castelo, Viana do Castelo, Portugal; e School of Sport Education, Tianjin University of Sport, 301617, Tianjian, China; f School of Physical Education, Zhengzhou University Headquarters, Henan, 450040, Henan, China

**Keywords:** Gymnastics, Physical fitness, Fatigue, Overreaching, Youth

## Abstract

Identifying indicators of non-functional overreaching during periods of increased training volume and/or intensity is particularly relevant for understanding the detrimental impacts incurred, as well as how these factors contribute to heightened injury risks among exposed athletes. This study aimed to compare the effects of a congested training period versus a standard training period on the strength levels and landing forces of female young aerobic gymnastics athletes. A prospective cohort study design was implemented, spanning four weeks. Fifty athletes (aged 16.2 ± 1.1 years old) at a trained/developmental level, competing at the regional level, were observed throughout the study. During two of these weeks (specifically weeks 2 and 3), half of the group was subjected to a congested training period consisting of six sessions per week (HTF), while the other half continued with their regular four sessions per week (STF). During each week of observation, participants underwent evaluation using the countermovement jump test (CMJ), squat jump test (SJ), and the leg land and hold test (LHT), with measurements taken on a force platform. The main outcomes repeatedly observed over the four weeks were CMJ peak landing force, CMJ peak power, SJ peak power, SJ maximum negative displacement, LHT time to stabilization, and LHT peak drop landing force. Significant interactions (time*group) were observed in CMJ peak power (p < 0.001), CMJ peak landing force (p < 0.001), SJ peak power (p < 0.001), SJ maximum negative displacement (p < 0.001), LHT time to stabilization (p < 0.001), and LHT peak drop landing force (p < 0.001). Furthermore, the results of the final assessment revealed significantly lower CMJ peak power (p = 0.008) and SJ peak power (p = 0.002) in the HTF group compared to the STF group. Additionally, significantly higher values of CMJ peak landing force (p = 0.041), SJ maximum negative displacement (p = 0.015), and LHT peak drop landing force (p = 0.047) were observed in the HTF group compared to the STF group. In conclusion, the increase in training frequency over two weeks significantly contributed to declines in neuromuscular power performance and peak landing forces. This indicates that intensified training periods may acutely expose athletes not only to performance drops but also to an increased risk of injury due to reduced capacity to absorb landing forces.

## 1 Introduction

Numerous athletes engage in extensive and challenge physical training regimens to enhance their performance [1]. However, an excess of physical training, insufficient recovery periods, and elevated overall stress levels can lead to temporary declines in performance [2]. This condition, commonly known as overreaching, presents a significant challenge for athletes aiming to reach peak performance [3]. Simultaneously, it can also affect critical factors linked to the causes of injury risk [4], by impairing immune function and reducing tissue repair capacities, thus compromising the athlete's ability to tolerate physical stressors and increasing the likelihood of injury.

At times, coaches deliberately induce overreaching before a recovery phase to stimulate a performance supercompensation [2]. This is achieved through intentional increases in volume or intensified training periods, such as shock microcycles or deliberate load progressions [5]. However, these consequent acute and deliberate declines in performance may have specific implications for key outcomes critical not only in meeting training demands but also in mitigating injury risks [6].

It is crucial to note that intensified and increased training volume can lead to two distinct overreaching concepts. Functional overreaching denotes a short-term training load increase resulting in temporary performance declines but eventual improvement post-recovery [7]. Conversely, non-functional overreaching entails a prolonged and excessive training load rise without adequate recovery, causing sustained performance declines and heightened injury risk, necessitating an extended recovery period spanning weeks to months [8].

The intensification of training or the increase in training volume is especially significant in sports, such as aerobic gymnastics, where athletes encounter a diverse range of circumstances involving jumps and landings, often performed unilaterally [9]. Ensuring appropriate physical fitness readiness is imperative to minimize the risk of injury [10]. This readiness entails maintaining balance, absorbing the impact of landings, and ensuring the correct biomechanical mechanisms are in place to apply force effectively, thus mitigating potential risks to athletes' safety [11].

Non-functional overreaching may induce alterations in neuromuscular function, including decreased motor unit recruitment and firing rates, which diminish the capacity of muscles to generate force efficiently [12,13]. Moreover, the repetitive high-impact nature of intense training can elevate landing forces during dynamic movements, predisposing athletes to greater risk of musculoskeletal injuries such as strains, sprains, and fractures [14]. These injuries often occur due to inadequate tissue adaptation and fatigue-induced alterations in movement mechanics, underscoring the importance of appropriate training periodization and recovery strategies to mitigate the detrimental effects of non-functional overreaching on strength and injury susceptibility [15]. For example, in rugby players, it was observed that muscular strength, power, and endurance decreased following overload training, indicating a state of non-functional overreaching possibly explained by increased muscle damage and a disruption in the anabolic-catabolic balance [16]. Similarly, in American football, high-volume conditioning drills were found to result in significant decrements in muscular strength and power [17].

Despite the importance of this topic, it is surprising that studies conducted in aerobic gymnastics or other gymnastics disciplines, which focus on understanding the impact of significant increases in load and/or training intensity, are not commonly observed. Understanding the risks and benefits of such strategies and adopting measures to mitigate negative impacts on performance and key strength outcomes are crucial for coaches and practitioners, especially in sports highly dependent on coping with high mechanical load impact and force absorption. Given this relevance, our study aimed to prospectively follow a cohort of young female aerobic gymnasts who were subjected to increased training volume, resulting from an elevated training frequency over a two-week period. Our objective was to compare the effects of this exposure against athletes who maintained their regular training routines, focusing on muscular strength and landing forces.

## 2 Methods

This study adhered to the Strengthening the Reporting of Observational Studies in Epidemiology (STROBE) [18] guidelines for reporting observational studies, specifically those involving cohort designs.

### 2.1 Study design

This study employed a prospective cohort design, where all participants were observed over four weeks with weekly evaluations. The researchers compared outcomes based on exposure to two different training frequencies in aerobic gymnastics: high training frequency (HTF: 6 training sessions per week; a total of 2295 min of accumulated training) and standard training frequency (STF: 4 training sessions per week; a total of 1845 min of accumulated training).

### 2.2 Setting

The study took place during the early phase of the sports season, one week after the return to training sessions. It was an observational study, meaning the researchers did not intervene in the training process, plans, or schedules of the clubs. However, the research team had prior knowledge of the periodization strategy, which involved increasing training frequency from 4 to 6 sessions per week for a group of the athletes (HTF). These athletes were those who could accommodate this increased training frequency into their schedules.

**Fig. 1:**
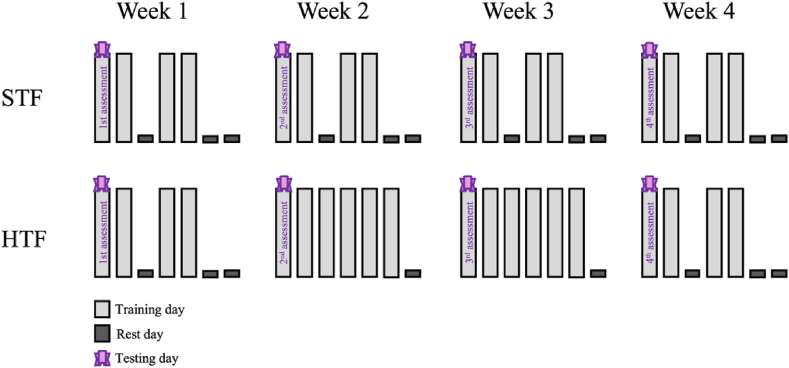
Study design. HTF: high training frequency; STF: standard training frequency.

During the first week, all athletes underwent assessments for muscular strength and power tests during their first training session of the week, following a 24-h rest period. After this initial week, one group of athletes was exposed to HTF while the other group underwent STF over the course of two consecutive weeks. By the fourth week, the HTF group reverted to the regular standard of 4 training sessions per week. Throughout all weeks, on the same day each week, the athletes were evaluated for their neuromuscular performance. Fig. 1 illustrates the study design.

### 2.3 Participants

A priori sample size estimation was performed for a repeated measures ANOVA with a within-between interaction involving 2 groups and 4 measurements. The analysis aimed for a power of 0.85, an effect size of 0.2, and a significance level of 0.05. This estimation was conducted using G*power software (version 3.1.9, Universität Düsseldorf, Germany), which recommended a sample size of 40 participants.

This study employed convenience sampling, wherein a regional club hosting aerobic gymnastics athletes granted researchers permission to conduct observations. The recruitment process involved verbal requests made to directors, coaches, and parents of the participants, aiming to ensure their participation. The eligibility criteria defined for inclusion in the study were as follows: (i) not missing any of the assessment moments and tests; (ii) not experiencing any injuries in the month prior to the study, and remaining injury-free or illness-free throughout the observation period; (iii) attending at least 90 % of all expected training sessions during the observation period; (iv) being female and having more than 2 years of experience as an aerobic gymnastics athlete; and (v) refraining from using any drugs or stimulant products during the observed period. Following the recruitment process, 57 athletes were identified and made themselves available to voluntarily participate in the study. However, after screening them against the eligibility criteria, 7 were excluded due to injuries sustained in the month prior to the study. Therefore, 50 eligible participants were included in the analysis. Out of these 50 participants, 24 took part in the HTF group, while 26 were in the STF group.

The 50 participants in this study were young female athletes (age: 16.2 ± 1.1 years; body mass: 52.9 ± 2.3 kg; height: 1.61 ± 0.03 m; experience: 6.1 ± 1.3 years), affiliated with a regional-level aerobic gymnastics team. They possessed a level of expertise categorized as trained/developmental, in line with the participant's classification framework, indicating a local-level representation, strong identification with the sport, and focused training aimed at competitive performance [19]. In the HTF group, the participants were aged 16.2 ± 1.2 years, with an average height of 1.60 ± 0.03 m and a body mass of 52.3 ± 2.8 kg. In the STF group, the participants were aged 16.3 ± 1.0 years, with an average height of 1.61 ± 0.03 m and a body mass of 53.5 ± 1.7 kg.

The study participants, including aerobic gymnastics athletes and their respective parents or legal guardians, were thoroughly briefed on the research protocol and its context. Prior to their voluntary involvement, legal guardians affirmed their consent by signing an informed consent document. Approval for this study was granted by the Ethics Committee of Tianjin Institute of Physical Education, with the reference code TJUS2024/016, ensuring adherence to the ethical principles outlined in the Declaration of Helsinki pertaining to research involving human subjects.

### 2.4 Independent variable

The independent variable considered in this study was the training frequency (HTF or STF) to which athletes were enrolled. The assignment to the groups was solely the responsibility of the club coaches and also depended on the athletes' availability to be assigned to the HTF group or not. The researchers had no intervention or decision-making role in this process; their sole responsibility was to identify those enrolled in a given group and then conduct evaluations over the four weeks.

During the four weeks, the HTF group accumulated 20 training sessions, while the STF group accumulated 16 training sessions. However, during the two weeks of load progression (2nd and 3rd weeks of observation), the HTF group had 12 training sessions, whereas the STF group had 8 training sessions. The athletes shared the same training context, with both groups undergoing identical training processes during regular sessions. However, in the case of HTF, additional sessions were not shared with STF, as they were supplementary sessions specifically tailored for focusing on increasing the competitive routines and sequences.

### 2.5 Training load monitoring

Throughout the analyzed training sessions, the athletes underwent daily monitoring using the Rate of Perceived Exertion (RPE), specifically utilizing Borg's CR10 scale [20]. The scale was administered individually approximately 30 min after the ending of each training session to prevent the terminal elements of an exercise session from unduly influencing the rating [21]. When prompted with the question “How intense was the training session?” athletes provided a score ranging from 0 to 10, referencing a support document containing verbal anchors identical to the original version [20]. These anchors were adapted to the native language of the participants to ensure clarity and accuracy of interpretation. Familiarization with the scale occurred in the week preceding the study's commencement, involving explanations of scale significance and verbal anchors, alongside practical application during training sessions not included in the subsequent analysis. Score recordings were consistently performed by the same researcher. Additionally, the duration of each training session was recorded, enabling multiplication of the RPE score by session duration in minutes to derive the session-RPE [22]. This measure was utilized to characterize athletes' exposure to training load over the four-week period and assess the impact of congested training weeks. The accumulated load per week (measured in arbitrary units, A.U.), which corresponds to the sum of all training load sessions for a given week, was calculated and used for further data treatment.

### 2.6 Assessment procedures

The assessments took place during the initial training session of each week, serving as the first step before the day's training session. Both groups and all conditions ensured a minimum rest period of 24 h. Assessments were conducted in a quiet room, individually. The room was maintained at a conditioned temperature of 23 °C with a relative humidity of 45 %. The assessments were consistently conducted by the same researchers in the afternoon. Participants were assessed individually in the same order, following a sequence of tests: (i) squat jump test (SJ); (ii) countermovement jump test (CMJ); and (iii) Land and hold test (LHT). Prior to the assessments, a standardized warm-up protocol was implemented, comprising 5 min of jogging in the gymnasium, followed by 5 min of lower-limb dynamic stretching targeting adductors, abductors, hamstrings, quadriceps, and gastrocnemius muscles, and concluding with 5 repetitions of vertical jumps and 5 repetitions of horizontal jumps. A 3-min interval was provided between the end of warm-up and the start of the tests. Additionally, a 5-min rest period was implemented between tests to mitigate potential fatigue effects.

### 2.7 Squat jump test (SJ)

The standard SJ (Squat Jump) test was administered. Participants began the test by assuming a squat position at a comfortable depth, with hands on hips and feet positioned comfortably. Upon the signal, participants were instructed to execute a powerful jump while keeping their hands on their hips and ensuring their knees remained extended during the flight phase.

During the initial week, participants completed one familiarization trial to ensure proper execution, with guidance from feedback provided by the researchers. Throughout all assessment days, participants performed two repetitions, with a 3-min rest period between each repetition. Measurements were taken using a force platform (ForceDecks, VALD, Brisbane, Australia). SJ peak power (N) and maximum negative displacement (cm) were recorded from the VALD ForceDecks software for each trial. The mean of the trials per session was then calculated for further data analysis.

### 2.8 Countermovement jump testing (CMJ)

The conventional CMJ test was conducted. Participants were instructed to begin in a standing position with their hands on their hips. Following a countdown, they initiated the movement by gradually descending into a comfortable squat position and then immediately transitioning into the ascending phase for the jump. Participants were instructed to maintain continuous movement from descent to ascent without any pauses. During the jump, they were instructed to keep their hands on the hips and knees extended and to land with both feet simultaneously on the platform.

During the initial week, participants underwent one familiarization trial to ensure proper execution, guided by feedback from the researchers. Throughout all assessment days, participants performed two repetitions, separated by 3 min of rest. Measurements were taken using a force platform (ForceDecks, VALD, Brisbane, Australia). CMJ peak power (W/kg) and landing force (N) were extracted from the VALD ForceDecks software for each trial. The mean of the trials per session was utilized for further data analysis.

### 2.9 Land and hold test (LHT)

The LHT (Landing Height Test) assesses both stability and how effectively an athlete manages landing impact forces. Participants began by standing on a 30 cm box, with the force platform positioned in front of it. Upon the signal, participants, with hands on hips, were instructed to step off the box with one foot leading, allowing the body to drop, and then land with both feet simultaneously, with specific emphasis on landing as smoothly as possible. Upon landing, they were instructed to maintain this position for 3 s.

During the initial week, participants completed one familiarization trial to ensure proper execution, with guidance from feedback provided by the researchers. Throughout all assessment days, participants performed two repetitions, with a 3-min rest period between each repetition. Measurements were taken using a force platform (ForceDecks, VALD, Brisbane, Australia). LHT time to stabilization (s) and landing force (N) were recorded from the VALD ForceDecks software for each trial. The mean of the trials per session was then calculated for further data analysis.

### 2.10 Statistical analysis

Prior to conducting inferential statistics, the normality of the sample distribution was assessed and confirmed using the Kolmogorov-Smirnov test (p > 0.05), while the assumption of homogeneity was verified through Levene's test (p > 0.05). Given the study's design, which involved two assessments across three groups, a mixed ANOVA was employed to explore interactions between time and groups. This analysis also included the calculation of partial eta squared ($\eta_{p}^{2}$). Thresholds for interpretation were established as follows [23]: values above 0.01 were considered small, those surpassing 0.06 were categorized as moderate, and values exceeding 0.14 were regarded as large. Additionally, post-hoc comparisons were conducted using the Bonferroni test. Statistical analyses were performed using JASP software (version 0.18.3, University of Amsterdam, The Netherlands), with a predetermined significance level of p < 0.05.

## 3 Results

Significant interactions (time*group) were observed in CMJ peak power (*F* = 126.855; *p* < 0.001; $\eta_{p}^{2}$ = 0.725), CMJ peak landing force (*F* = 137.910; *p* < 0.001; $\eta_{p}^{2}$ = 0.742), SJ peak power (*F* = 120.492; *p* < 0.001; $\eta_{p}^{2}$ = 0.715), SJ maximum negative displacement (*F* = 55.048; *p* < 0.001; $\eta_{p}^{2}$ = 0.534), LHT time to stabilization (*F* = 36.375; *p* < 0.001; $\eta_{p}^{2}$ = 0.431), and LHT peak drop landing force (*F* = 137.763; *p* < 0.001; $\eta_{p}^{2}$ = 0.742).

Table 1 presents the descriptive statistics of the outcomes analyzed in both HTF and STF groups. No significant differences (*p* > 0.05) between groups were found at baseline (1st assessment), 2nd, and 3rd assessment moments in any of the analyzed outcomes, namely, CMJ peak power, CMJ peak landing force, SJ peak power, SJ maximum negative displacement, LHT time to stabilization, and LHT peak drop landing force.

**Table 1:** Descriptive statistics (mean and standard deviation) of the outcomes across the four assessment moments for both groups: high training frequency (HTF) and standard training frequency (STF).

Outcome	HTF (n = 24)	STF (n = 26)	Between-group
CMJ peak power (W/kg)			
1st assessment	31.8 ± 4.3^c^^,^^d^	31.4 ± 5.0	*p* = 0.765
2nd assessment	31.7 ± 4.3^c^^,^^d^	31.3 ± 5.2^c^^,^^d^	*p* = 0.758
3rd assessment	30.4 ± 4.2^a^^,^^b^^,^^d^	31.7 ± 5.5^b^^,^^d^	*p* = 0.361
4th assessment	28.2 ± 4.0^a^^,^^b^^,^^c^	32.1 ± 5.6^b^^,^^c^	*p* = 0.008*
CMJ peak landing force (N)			
1st assessment	1438.0 ± 444.7^c^^,^^d^	1449.2 ± 393.9^c^^,^^d^	*p* = 0.925
2nd assessment	1439.1 ± 440.6^c^^,^^d^	1452.3 ± 407.9^c^^,^^d^	*p* = 0.913
3rd assessment	1536.0 ± 479.9^a^^,^^b^^,^^d^	1407.9 ± 388.4^a^^,^^b^^,^^d^	*p* = 0.303
4th assessment	1630.5 ± 519.5^a^^,^^b^^,^^c^	1362.4 ± 375.3^a^^,^^b^^,^^c^	*p* = 0.041*
SJ peak power (W/kg)			
1st assessment	7.08 ± 0.98^c^^,^^d^	7.01 ± 1.26^d^	*p* = 0.823
2nd assessment	7.09 ± 0.96^c^^,^^d^	7.01 ± 1.22^d^	*p* = 0.796
3rd assessment	6.75 ± 0.90^a^^,^^b^^,^^d^	7.09 ± 1.25^d^	*p* = 0.287
4th assessment	6.21 ± 0.82^a^^,^^b^^,^^c^	7.24 ± 1.32^a^^,^^b^^,^^c^	*p* = 0.002*
SJ maximum negative displacement (cm)			
1st assessment	29.8 ± 9.4^c^^,^^d^	27.3 ± 7.6	*p* = 0.302
2nd assessment	30.2 ± 9.5^c^^,^^d^	27.3 ± 7.6^d^	*p* = 0.246
3rd assessment	31.1 ± 10.0^a^^,^^b^^,^^d^	27.2 ± 7.7^d^	*p* = 0.125
4th assessment	32.9 ± 10.6^a^^,^^b^^,^^c^	26.5 ± 7.3^b^^,^^c^	*p* = 0.015*
LHT time to stabilization (s)			
1st assessment	0.42 ± 1.15^c^^,^^d^	0.43 ± 0.13	*p* = 0.946
2nd assessment	0.43 ± 0.16^c^^,^^d^	0.43 ± 0.14	*p* = 0.918
3rd assessment	0.46 ± 0.16^a^^,^^b^^,^^d^	0.43 ± 0.15	*p* = 0.573
4th assessment	0.49 ± 0.17^a^^,^^b^^,^^c^	0.42 ± 0.14	*p* = 0.186
LHT peak drop landing force (N)			
1st assessment	1112.4 ± 390.2^c^^,^^d^	1178.3 ± 260.0^c^^,^^d^	*p* = 0.483
2nd assessment	1131.0 ± 400.7^c^^,^^d^	1181.4 ± 254.5^c^^,^^d^	*p* = 0.596
3rd assessment	1209.4 ± 427.2^a^^,^^b^^,^^d^	1144.1 ± 251.5^a^^,^^b^^,^^d^	*p* = 0.509
4th assessment	1296.3 ± 457.1^a^^,^^b^^,^^c^	1088.1 ± 240.4^a^^,^^b^^,^^c^	*p* = 0.047*

a Significantly different from the 1st assessment.

b Significantly different from the 2nd assessment.

c Significantly different from the 3rd assessment.

d Significantly different from the 4th assessment.

However, significant differences were found between groups at the 4th assessment moment. Specifically, significantly lower CMJ peak power (*p* = 0.008) and SJ peak power (*p* = 0.002) were observed in HTF compared to STF. Additionally, significantly higher values of CMJ peak landing force (*p* = 0.041), SJ maximum negative displacement (*p* = 0.015), and LHT peak drop landing force (*p* = 0.047) were observed in HTF compared to STF. No significant differences were found in LHT time to stabilization (*p* = 0.186).

**Fig. 2:**
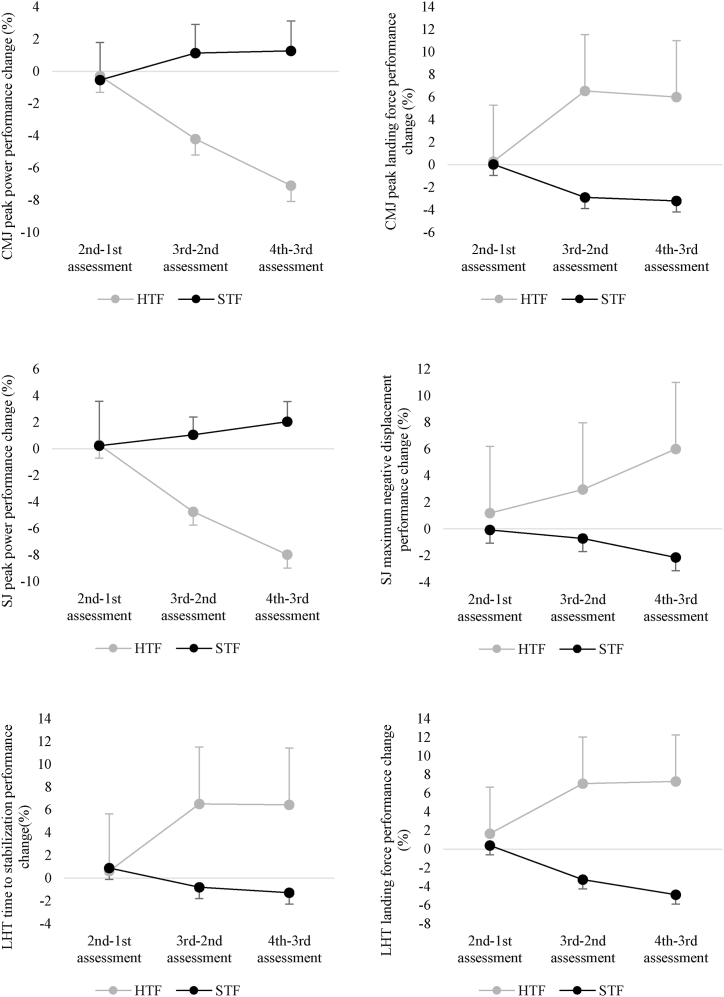
Change in outcomes analyzed (%) for each high training frequency (HTF) and standard training frequency (STF) groups.

Fig. 2 shows the percentage differences observed over the weeks for each group analyzed. The within-HTF analysis revealed a significant decline in the fourth assessment compared to the baseline assessment, the second and third assessments showed significant declines in CMJ peak power (p < 0.001), CMJ peak landing force (p < 0.001), SJ peak power (p < 0.001), SJ maximum negative displacement (p < 0.001), LHT time to stabilization (p < 0.001), and LHT peak drop landing force (p < 0.001). From the baseline to the second assessment, which corresponds to a standard week of training, the HTF showed no significant variations (p > 0.05) in any of the outcomes analyzed.

The within-STF analysis revealed no significant difference in CMJ peak power between the baseline and the other assessment moments (p > 0.05). However, the third assessment showed significant higher results than both the second (p = 0.046) and the fourth (p = 0.003). Regarding CMJ landing force, the fourth assessment was significantly better than the baseline (p < 0.001), the second assessment (p < 0.001), and the third assessment (p < 0.001). In terms of SJ peak power, the fourth assessment showed the best performance compared to the baseline (p = 0.003), the second assessment (p < 0.001), and the third assessment (p < 0.001). For SJ maximum negative displacement, the best performance was observed in the fourth assessment compared to the second assessment (p = 0.025) and the third assessment (p = 0.004), although no significant differences were observed with the baseline (p = 0.112). Regarding LHT time to stabilization, no significant differences were observed between assessment periods (p > 0.005). Finally, the LHT peak drop landing force had the significantly better score in the fourth assessment compared to the baseline (p < 0.001), the second (p < 0.001), and the third assessments (p < 0.001).

**Fig. 3:**
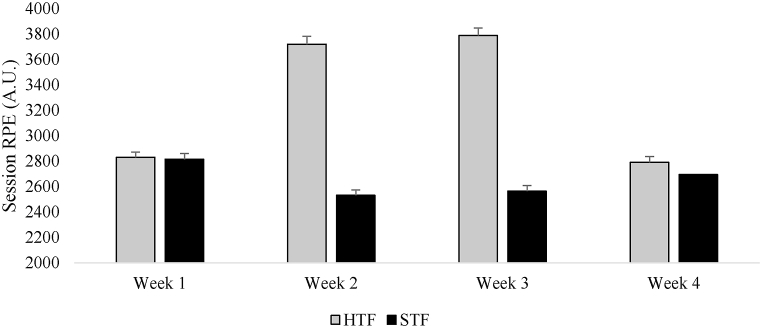
Descriptive statistics (mean ± standard deviation) of the weekly accumulated session rate of perceived exertion (session RPE) over the four weeks analyzed for both the high training frequency (HTF) and standard training frequency (STF) groups.

Fig. 3 shows the descriptive statistics of the accumulated weekly training load over the four weeks analyzed. It was observed that in weeks 1 and 4, the mean differences between the groups were small (week 1: HTF 2830 ± 41 A.U. vs. STF 2815 ± 39 A.U.; week 4: HTF 2790 ± 46 A.U. vs. STF 2694 ± 45 A.U.), while the greater differences occurred in weeks 2 and 3 (week 2: HTF 3719 ± 61 A.U. vs. STF 2531 ± 46 A.U.; week 3: HTF 3789 ± 60 A.U. vs. STF 2564 ± 42 A.U.). It is also important to highlight that from the first to the second week, the HTF group increased their training load by 31.4 % due to the increase in training frequency from 4 to 6 sessions per week. In weeks 1 and 4, both groups had an accumulated training duration of 450 and 460 min, respectively. In weeks 2 and 3, the HTF group had accumulated training durations of 685 and 700 min, respectively, while the STF group had 460 and 475 min, respectively.

## 4 Discussion

Our study revealed that athletes exposed to a 30 % increase in training load, compared to those maintaining their training frequency and load, experienced significant declines in neuromuscular performance and the ability to absorb landing forces. These effects were significantly observed after one week and continued to decline after two weeks of exposure. This evidence is particularly relevant in a sport like aerobic gymnastics, where athletes frequently perform powerful movements, jumps, and landings. The decline in performance not only reduces their ability to meet session demands but also may increases the risk of injury due to intensified landing forces.

In our study, we observed that landing forces in both the CMJ and LHT tests increased by approximately 7 % in the HTF group after the first week of intensified training. These forces continued to rise by 6 % and 7 % after the second week, respectively. The increase in landing forces within the HTF group can be attributed to physiological mechanisms, primarily non-functional overreaching [24] and neuromuscular fatigue [25], which directly impact performance. Non-functional overreaching typically results from an imbalance between training intensity and recovery [26], leading to accumulated central and peripheral fatigue that may impairs the neuromuscular system's optimal function [27].

Central fatigue can reduce motor unit recruitment and alter firing patterns [28], while peripheral fatigue can diminish a muscle's capacity to generate and withstand forces efficiently [29]. In the HTF group, with six training sessions per week and inadequate recovery, these forms of fatigue likely contributed to the increased landing forces during the CMJ and LHT tests. Fatigued muscles may have a reduced ability to attenuate impact forces, resulting in higher ground reaction forces upon landing [30].

This increase in landing forces is particularly concerning in aerobic gymnastics, a discipline that requires precise and controlled landings to minimize injury risk and optimize performance [31]. The heightened forces observed in the HTF group suggest a compromised ability to sustain landing forces, potentially increasing the likelihood of injuries [32].

In addition to the significant increases in landing forces observed in the HTF group, these athletes experienced significant declines in neuromuscular performance, as evidenced by decreased peak power outputs during CMJ and SJ tests. These declines became significantly different from those observed in the STF group. This aligns with a previous study showing that intensified and congested military field training significantly impairs neuromuscular performance as measured by jump tests [33]. Once again, this temporary decline could be attributed to a state of non-functional overreaching [24]. While intensified training volume may contribute to performance enhancement after tapering, if not properly managed, non-functional overreaching can lead to transient declines in performance before subsequent improvements occur [3].

One hypothesis to explain the decline in neuromuscular performance following intensified training frequency is the accumulation of acute fatigue without adequate recovery [34]. Over the two weeks of increased accumulated training load, there may have been a buildup of cumulative fatigue, resulting in decreased neuromuscular function. This acute fatigue can manifest as reduced peak power outputs during explosive movements such as those involved in CMJ and SJ tests [35]. Possibly, the increased training volume associated with a compromised recovery process may have disrupted the neuromuscular coordination and motor unit recruitment patterns necessary for generating maximal force during explosive movements [36]. Peripheral fatigue may also have played a role, with the intensified training frequency leading to glycogen depletion, metabolic disturbances, and impaired muscle contractile function [37].

While our study provides valuable insights into the effects of intensified training load on neuromuscular performance and landing forces in aerobic gymnasts, some limitations should be acknowledged. Firstly, our study focused solely on short-term effects over a two-week period. Longitudinal studies with extended follow-up periods could provide a more comprehensive understanding of how these effects evolve over time and whether they persist or attenuate with continued training. Moreover, by conducting a longitudinal study with assessment points following the increased volume and congested period, we could understand the duration of impairment and determine whether performance returns to expected levels in the subsequent weeks. Additionally, the study sample consisted of female young aerobic gymnasts, limiting the generalizability of the findings to other sports or athletic populations. Future research could explore how different training intensities and frequencies impact neuromuscular performance and landing forces across various sports disciplines. Moreover, while we speculated on the physiological mechanisms underlying the observed effects, further investigation using direct measures of fatigue, such as electromyography or biochemical markers, could provide deeper insights into the underlying mechanisms. Furthermore, our study did not assess potential confounding variables such as sleep quality, nutrition, or psychological stress, which could influence both training adaptation and neuromuscular performance. Future studies could incorporate comprehensive monitoring of these factors to better elucidate their contributions to training outcomes.

Despite the study limitations, the findings from our study emphasize the importance of carefully managing training load in aerobic gymnasts to optimize performance and reduce injury risk. Introducing a 30 % increase in training load over a two-week period resulted in significant declines in neuromuscular performance and increased landing forces, highlighting the potential negative consequences of abrupt training volume increase. Coaches and athletes should prioritize adequate recovery periods to prevent the accumulation of fatigue and optimize training adaptations. Moreover, individualized training plans considering athlete characteristics and monitoring training load-response relationships can help mitigate the detrimental effects of intensified training. For example, it is important for coaches to implement both internal and external load measures during training sessions, such as heart rate sensors or perceived exertion scales, as well as microelectromechanical sensors to analyze the mechanical demands. Additionally, it is recommended to conduct weekly tests to measure fatigue and physical readiness, such as countermovement jump or isometric mid-thigh pull tests, which are sensitive to minor changes in neuromuscular readiness and can guide coaches on the athletes' recovery status. By incorporating these principles and strategies into training programs, coaches can foster long-term athlete development while minimizing the risk of injury associated with high training loads. Ultimately, our findings emphasize the importance of a strategic and holistic approach to training management in optimizing athletic performance in aerobic gymnastics.

## 5 Conclusions

The current cohort study revealed that young female aerobic gymnastics athletes, when exposed to an increased volume of training due to a rise in weekly training frequency, are subject to non-functional overreaching and declines in neuromuscular performance. This non-functional overreaching acutely and significantly reduces neuromuscular power outcomes and increases landing forces in jump movements, which are frequent in this sport. Therefore, it is particularly relevant to pay attention to intensified training periods, as non-functional overreaching may potentially expose athletes to a higher risk of injury due to increased landing forces. Monitoring the acute impact of accumulated training, utilizing systems for assessing neuromuscular readiness, and controlling or mitigating the impact through appropriate recovery strategies and adjustments in training planning are recommended actions to manage periods of significant increases in training volume and/or intensity.

## Data availability

The data is available upon reasonable request to the corresponding author.

## Funding

There is no funding to report.

## Human ethics and consent to participate declarations

The study adheres to the ethical standards outlined in the Declaration of Helsinki, and the study protocol received approval from the Ethics Committee of Tianjin Institute of Physical Education under code number TJUS2024/016. The participants and their legal guardians have signed a freely given informed consent to participate in the study and to have the study results anonymously disclosed.

We confirm that consent/assent was obtained from minor(s) in addition to parental/guardian consent.

## Consent for publication

Not applicable.

## CRediT authorship contribution statement

**Dong Ma:** Writing – review & editing, Writing – original draft, Methodology, Investigation, Formal analysis, Data curation, Conceptualization. **Kezhan Zhao:** Writing – review & editing, Writing – original draft, Investigation. **Rui Miguel Silva:** Writing – review & editing, Writing – original draft. **Ke Wang:** Writing – review & editing, Writing – original draft, Methodology, Investigation. **Qi Xu:** Writing – review & editing, Writing – original draft. **Zijian Zhao:** Writing – review & editing, Writing – original draft, Supervision, Methodology, Investigation, Formal analysis, Data curation, Conceptualization.

## Declaration of competing interest

The authors declare that they have no known competing financial interests or personal relationships that could have appeared to influence the work reported in this paper.
